# When forensic psychiatry clashes with jurisdiction: nemesis and challenges of a patient suffering from glioblastoma

**DOI:** 10.1192/bjo.2026.11022

**Published:** 2026-03-27

**Authors:** Ambra D’Imperio, Didier Delessert, Marcello Ienca, Georg Starke

**Affiliations:** Institute of History and Ethics in Medicine, Technical University of Munich, Munich, Germany; University Clinic of Forensic Psychiatry, University of Bern, Bern, Switzerland; Department of Psychiatry, Prison Medicine and Psychiatry Service, Vaud University Hospital Center, Lausanne, Switzerland; Freiburg Institute for Advanced Studies, Albert-Ludwigs-University of Freiburg, Freiburg, Germany

**Keywords:** Forensic psychiatry, palliative care, arts psychiatry, psychiatry and law, assisted suicide

## Abstract

This case study explores the interaction of brain pathology, criminal behaviour and art in forensic psychiatry through the case of a 68-year-old man exhibiting neuropsychiatric symptoms and delusions. His progressive cognitive and emotional deterioration led to aggressive behaviour, threats towards colleagues and family and allegations of violent and sexual assault. After months of his refusing treatment, magnetic resonance imaging revealed a grade IV glioblastoma. Despite the terminal diagnosis, he was placed in a forensic acute psychiatric unit while under prosecutorial investigation, because his actions remained criminally relevant. In this restrictive setting, he turned to drawing as his primary coping strategy. His artwork offered both an outlet for suffering and a means of transcending a situation characterised by severe illness, legal deprivation of liberty and existential despair. The case illustrates the benefit of creative expression when medical and legal circumstances appear intractable. It also raises ethical and forensic concerns, including impaired culpability due to amygdala and prefrontal damage, refusal of care, thoughts of physician-assisted suicide and the attribution of legal responsibility.

The landmark cases of Phineas Gage and Charles Whitman continue to resonate in textbooks of neuroscience and psychiatry. Phineas Gage, who sustained a severe prefrontal cortex injury in 1848, subsequently exhibited profound behavioural changes, including impulsivity, irritability and disinhibition. More than a century later, Charles Whitman’s violent trajectory culminated in the killing of 17 people in 1966 after a tumour grew in his temporal lobe. These cases are not merely historical curiosities: they continue to frame contemporary debates on how neuropathology can profoundly alter human behaviour. Both cases have also fuelled extensive scholarly discourse regarding the potential cortical localisation of criminal behaviours, and on the extent to which such alterations should mitigate criminal responsibility.^1,2^

In recent decades, ample research has been dedicated to understanding the neural correlates of emotion regulation and impulse control. The amygdala and ventrolateral prefrontal cortex have been identified as key regions implicated in action planning.^3^ Furthermore, the dorsomedial prefrontal cortex and ventromedial prefrontal cortex have likewise been implicated in higher-order regulation of emotional and cognitive processes, whereas the amygdala and hippocampus have been recognised as central to the modulation of emotional responses. Lesions of the frontal lobe can significantly disrupt behavioural equilibrium, with the dorsolateral frontal lobe being critical for action inhibition and the anterior cingulate cortex (ACC), orbitofrontal cortex and amygdala contributing to error detection, reward-processing and emotional decision-making linked to impulsivity.^4,5^ These neural mechanisms are not only of academic interest: in forensic psychiatry, they directly inform judgements about culpability, risk assessment and the boundaries of legal responsibility.

Current approaches to brain dysfunction in forensic psychiatry often fall into two principal camps. One starts with neurobiological dysfunction, aiming to identify distinct brain regions responsible for behavioural disturbances.^6^ The other emphasises an interpretative psychological analysis, examining behaviour first before exploring possible connections to any underlying organic abnormalities.^7^ Despite advances in both domains, legal systems frequently struggle to integrate neuroscientific findings into adjudicating criminal responsibility. Although contemporary research avoids strict causal and deterministic explanations of criminality, there is broad consensus that criminal conduct is frequently precipitated by specific contributory factors such as organic brain injury, substance use or neuro-inflammation, rather than being the result of a single, deterministic pathway.^8^ Furthermore, investigations into psychopathology have consistently failed to identify a unified causal model, underscoring the enduring significance of self-determination, intentionality and responsibility.^9^ These elements, combined with forensic psychiatric expertise, form the foundation of what is often termed forensic truth.

Alongside these neuroscientific and forensic perspectives, the role of art in psychiatry has a long history. In the early 20th century, Hans Prinzhorn, art historian and physician working at that time in Heidelberg, systematically collected the artistic productions of patients in psychiatry, most of whom had never previously expressed themselves artistically.^10^ Around the same time, Walter Morgenthaler in Bern documented the life and works of his patient Adolf Wölfli, whose prolific artistic output remains a landmark in both psychiatric history and outsider art.^11^ These contributions and others resulted in art brut becoming known as a powerful mode of expression for patients whose voices might otherwise be marginalised by illness or institutionalisation. Within psychiatry, artistic expression has since been recognised not only as a diagnostic or therapeutic tool but also as a means of preserving individuality and agency in the contexts of suffering, coercion or confinement.

Forensic psychiatry can be a particularly important area for such preservation. Although evaluations typically proceed under the presumption of innocence,^12^ this presumption is sometimes further supported by findings that indicate partial or complete exemption from criminal responsibility owing to an underlying organic cause. However, what should be the course of action if the defendant is found to suffer not only from an organic disease affecting their behaviour, but also from a malignant tumour carrying a life expectancy shorter than the duration of the legal proceedings? We here present such a case in which the patient, in addition to neuropsychiatric and forensic complexities, resorted to drawing as a primary means of coping, reflection and communication. His artwork, created under conditions of illness, confinement and prosecution, offers a rare perspective on the intersection of neuropathology, criminal behaviour and art in forensic psychiatry.

## Method

### Case collection

This article is based on a clinical case encountered by the first author in her professional capacity as a psychiatrist in Switzerland. The patient provided written informed consent in accordance with the hospital’s guidelines. Identifiable personal data have been removed or modified to ensure confidentiality and anonymity. No additional research intervention was carried out, and therefore no formal ethics approval was required.

The authors assert that all procedures contributing to this work comply with the ethical standards of the relevant national and institutional committees on human experimentation, and with the Declaration of Helsinki of 1964 as revised in 2024.

### Medical history

The patient, a 68-year-old retired male living alone, had led a varied professional life, working in nursing and carpentry before qualifying as a primary school teacher in 2009. His career was interrupted by medical issues in 2008–2009, leading to part-time work until 2012, when he again went on medical leave. Following multiple assessments, he was granted a full disability pension in 2018. His medical history initially included generalised chronic pain and hypertension, manageable with medication, and there was no family history of genetic or neurodegenerative diseases.

In April 2021 the patient’s condition worsened markedly, with seizures and escalating behavioural changes including impulsivity, irritability and resistance to medical, social and legal interventions. He exhibited suspicion towards the medical system and refused prescribed medications. His behaviour became increasingly erratic, exemplified by incoherent and aggressive communications and fleeing his home town to avoid perceived legal consequences. Family members expressed growing concerns as his social functioning deteriorated, culminating in paranoid suspicions of conspiracy and the appointment of a conservator to manage his social and financial affairs. In 2022, following a bicycle accident and law enforcement involvement, he was admitted under emergency placement to the local university hospital as ordered by regional law enforcement authorities. Upon admission he exhibited psychomotor agitation and clear cognitive impairment. He was unable to distinguish trivial from essential matters, and his speech was marked by thematic leaps and persistent delusions involving conspiracies by political and judicial authorities.

Between 2022 and 2024 the patient’s neuropsychological condition deteriorated further. Relatives and former colleagues noted marked behavioural changes. His thinking became dominated by delusional beliefs, including repeated accusations that the treatment team was conspiring with political and judicial entities and engaged in tax evasion. He frequently expressed these delusions through emails and letters, and made both written and verbal threats against his family and colleagues.

During admission to the geriatric psychiatric ward he exhibited physical aggression, injuring a nurse, which necessitated transfer to a secure intensive treatment room. Sedatives were administered without his consent as part of a mandatory treatment. Although this intervention alleviated his acute agitation, his chronic delusional thinking and formal thought disorder persisted. Despite this, the patient continued to refuse most treatments. He consistently rejected both antipsychotic medication and instrumental examinations, including cerebrospinal fluid analysis, magnetic resonance imaging (MRI) and an electroencephalogram, and declined to participate in clinical assessments such as the Structured Clinical Interview for DSM-5, Montreal Cognitive Assessment, Beck Depression Inventory and Positive and Negative Symptoms Severity Scale. Blood tests revealed deficiencies in vitamins B12 and D and a borderline prolonged clotting time. Despite medical advice to adjust his antihypertensive medication due to persistently high blood pressure, he refused, citing his opposition to collaborating with physicians and, indirectly, with the judicial system which had, following several complaints, opened a penal investigation. The persistent refusal of treatment, and his continued adherence to delusional beliefs, underscored the diagnostic complexity of his condition, with differential considerations including severe depression, bipolar disorder and psychotic decompensation due to a neurodegenerative process, or to a chronic delusional disorder. Substance abuse was ruled out. During this period of neuropsychological distress, the patient committed several offences, notably threats and aggression towards third parties, and was also accused of sexual assault against a woman.

Clinical evaluation suggested impairments probably attributable to the right frontal lesion, primarily involving emotional regulation and personality changes and characterised by impulsivity, disinhibition and reduced social appropriateness. The clinical presentation was consistent with a frontal lobe syndrome, including impaired emotional inhibition with episodes of irritability, anger and heightened excitement, as well as difficulties in understanding others’ perspectives and a pronounced oppositional attitude towards the medical system. Behavioural alterations included occasional psychomotor agitation. Impulsivity played a central role, contributing to social disinhibition and disturbances in interpersonal boundaries, including inappropriate interpersonal approaches and misogynistic attitudes towards female nursing staff. Executive dysfunction was also evident, particularly in impaired judgement, reduced cognitive flexibility and diminished capacity to regulate his behaviour in accordance with social norms, resulting in functionally significant impairment of interpersonal and institutional functioning. These manifestations are characteristic of right frontal lobe dysfunction, which is typically associated with impulsive, disinhibited and socially inappropriate behaviours, in contrast to the apathetic and language-dominant deficits more commonly observed in left frontal lobe involvement. Overall, the severity of impairment was clinically moderate to severe, predominantly affecting behavioural and executive control, although basic orientation and global cognitive functioning remained relatively preserved.

Following prolonged resistance, he finally agreed to undergo MRI in 2024, resulting in the diagnosis of a right temporal glioblastoma, confirmed by biopsy as wild-type IDH of WHO grade IV. Neuro-radiological assessment described a heterogeneously contrast-enhancing mass centred in the right middle frontal gyrus, containing necrotic cores and surrounded by extensive vasogenic oedema with irregular infiltrative borders extending towards subcortical insular regions. The lesion produced a marked local mass effect on adjacent frontal networks implicated in executive control, behavioural inhibition and social regulation, providing a plausible neuro-anatomical substrate for the patient’s progressive disinhibition, impaired judgement and personality change.

### Forensic and criminal history

The patient had faced multiple criminal charges over the course of several years. Some of these charges, such as threats, defamation and slander against family members, colleagues and political officials, had already emerged in 2022. At that time, an initial MRI did not reveal any neurological or neurodegenerative conditions, leaving a primary psychiatric disorder as the most plausible explanation. As the patient’s condition deteriorated between 2022 and 2024, additional allegations accumulated, including verbal and physical threats, minor bodily harm and allegations of sexual coercion. This period of escalating behavioural dysregulation culminated in an indictment and forced hospitalisation in 2024, during which a WHO grade IV glioblastoma was diagnosed.

During these legal proceedings in 2024, two psychiatric expert reports were produced. The first evaluation, conducted shortly before the diagnostic work-up confirming the presence of glioblastoma, diagnosed a personality disorder with paranoid delusional features. The patient subsequently appealed against this conclusion, stating that he was not experiencing any psychotic disorder or related delusional symptoms. Following MRI and histopathological confirmation of a right frontal glioblastoma, a second psychiatric expert opinion excluded a primary psychiatric disorder and instead diagnosed secondary personality change according to the new ICD-11 classification 6E68,^13^ previously diagnosed as F06 (‘Other mental disorders due to brain damage and dysfunction and to physical disease’, ICD-10). This later opinion attributed the behavioural disturbances directly to the tumour and thereby substantially altered the forensic interpretation of criminal responsibility. In the subsequent legal evaluation, this tumour-based opinion formed the primary basis for determining the patient’s degree of criminal responsibility.

Under Swiss criminal code, criminal responsibility can be classified as either mild, moderate or highly impacted, reflecting impairments in cognitive and volitional capacities that may result in partial or complete criminal irresponsibility.^14^ This forensic assessment is legally distinct from civil law decision-making capacity and is determined specifically in relation to the criminal act under examination. In this patient, both capacities were affected by the right frontal lesion and subsequent personality changes, making it difficult to establish an exclusive causal link between the lesion and the cognitive component of criminal responsibility. Both volitional and cognitive components were altered, and the patient was found to be fully irresponsible. Under the Swiss legal framework, such a finding typically shifts the judicial response from punitive sentencing towards therapeutic or protective forensic measures.^15^

Regarding legal capacity, the patient had access to legal representation in accordance with Swiss criminal procedure yet chose to defend himself during the proceedings and did not appoint legal counsel. Forensic assessment nevertheless concluded that he retained sufficient procedural capacity to understand the nature of the legal process, articulate his position and participate meaningfully in his defence. This simultaneous coexistence of preserved procedural legal capacity with markedly impaired criminal responsibility represented a central medico-legal tension in the case.

**Fig. 1:**
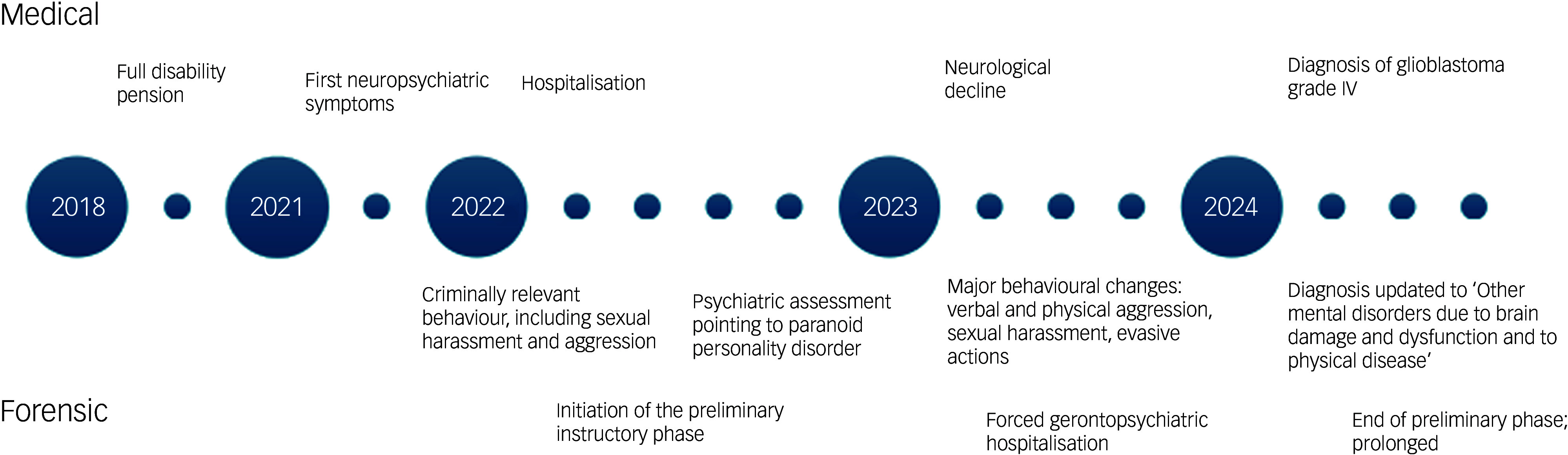
Overview of key steps in the patient’s medical and forensic history over 6 years.

At the time of admission to the acute forensic psychiatric ward, the patient was held in pre-trial detention under judicial order for several offences, including defamation, threats and sexual assault. His initial acts of physical aggression, directed towards both family members and a nurse, led to his psychiatric confinement and precipitated legal proceedings. During this period he displayed complete non-cooperation with medical staff, refusing all prescribed medications and treatments. Following the diagnosis of glioblastoma in 2024, his stance towards the medical team notably shifted. While he continued to refuse medical interventions, such as pharmacotherapy, he became more cooperative, participating in psychotherapy and music therapy sessions. An overview of key events in the patient’s medical and forensic history is provided in Fig. 1.

In summary, the patient’s trajectory progressed from emerging behavioural disturbance in 2021 to escalating criminal allegations between 2022 and 2024, and finally to the diagnosis of a malignant frontal brain tumour in 2024, highlighting complex questions about the role of brain disease in the assessment of his criminal responsibility.

### Artistic expression offering coping and resilience

Throughout his treatment the patient declined all psychopharmacological interventions, such as selective serotonin reuptake inhibitors, to improve mood and provide analgesic benefits, with the single exception of risperidone, which he valued for its sedative properties. Unexpectedly the patient refused antidepressants, citing the development of alternative coping mechanisms. He presented his psychotherapist with drawings created during his prolonged detention in prison and the forensic psychiatric ward. Supported by psychiatrists and psychologists, the patient engaged in art therapy as part of supportive psychotherapy, which, through a cognitive–behavioural approach, became his primary means of coping with the illness, the slow legal process and the constraints of the jurisdictional framework. The patient’s drawings were reviewed within this clinical context by the attending mental health professionals, including psychiatrists and psychotherapists, who reviewed the drawings primarily as expressions of subjective experience and coping. No independent expert assessment in the field of art therapy or outsider-art analysis was undertaken; accordingly, the artwork is presented here in an illustrative and phenomenological rather than diagnostic capacity.

In this sense, the patient’s artistic expression can be seen from a dual angle. Clinically, the drawings functioned as a medium for emotional regulation, autobiographical meaning-making and symbolic communication with the treatment team in the context of limited verbal engagement. From a forensic perspective, they also represented an attempt to reinterpret the legal narrative surrounding the alleged offences and to assert personal agency within conditions of detention and impending death. As such, the patient’s artwork operated simultaneously as therapeutic resource, existential expression and indirect dialogue with the medico-legal process.

**Fig. 2:**
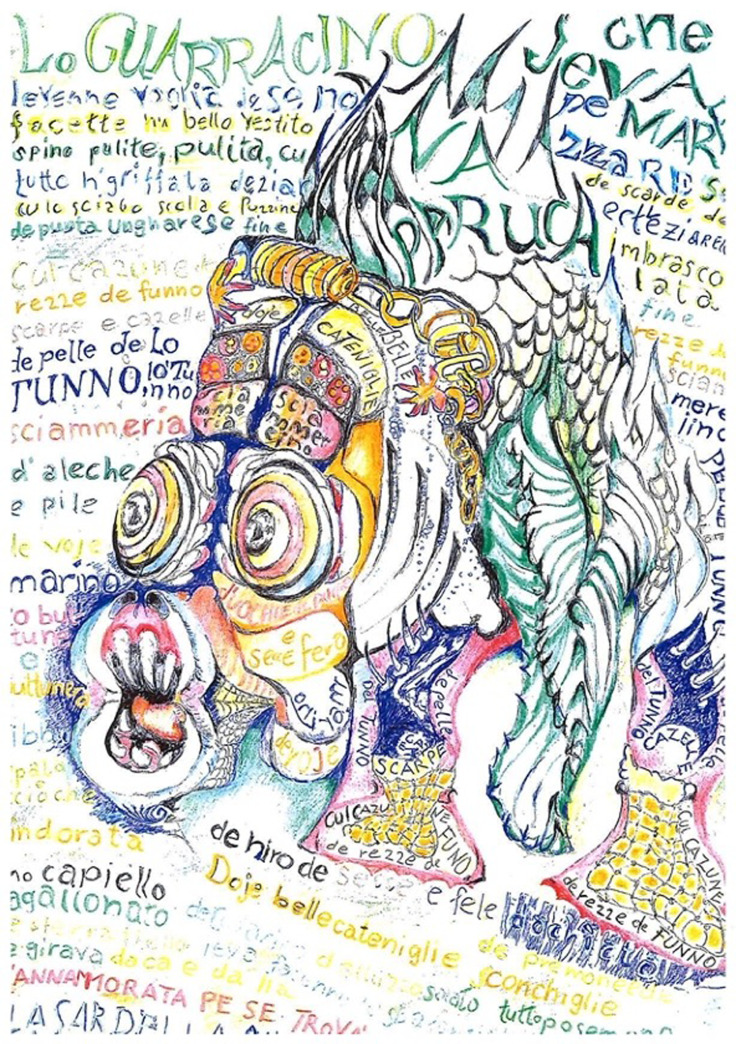
Il Guarracino.

The patient described humour as central to his worldview, and explained that his artwork depicted the ‘joke that society has played on him’. In this context, he referred to Neapolitan folklore, particularly the character ‘Guarracino’. As narrated in an 18th-century Neapolitan tarantella, the guarracino, a small Mediterranean fish, falls in love with an anchovy, triggering violent struggles among various involved fish due to love, malice and misunderstanding. The patient likened himself to this character, portraying his circumstances as the outcome of a tragic ‘misunderstanding’ and vehemently denying the sexual offences alleged by the prosecutor. These symbolic representations functioned as artistic outlets, allowing the patient to convey his disillusionment with society (Fig. 2).

In addition to psychiatric therapy, the patient accessed spiritual guidance from a theologian during his stay in the acute forensic psychiatric ward. Discussions centred around the meaning of death, with particular focus on scenarios involving potential death in the hospital. Topics included the possibility of suicide in a depressive state and the legal option of accessing physician-assisted suicide (PAS) in Switzerland. The patient’s stance was unequivocal: he meticulously planned his funeral rites, including musical selections and the projection of his drawings, while declaring his determination to live out his remaining months combating both his brain tumour and the constraints of forensic detention. Although he expressed theoretical agreement with the procedure of assisted suicide, he rejected the idea for himself, framing it as a capitulation to the system and thus as personal defeat. He argued that he had not yet endured sufficient suffering to die ‘heroically’, and did not want to leave this world as a ‘victim’ of his intractable situation, which he described as his ‘nemesis’. Above all, the patient wanted to share his story and his artwork to aid others experiencing similar situations.

From a psychotherapeutic perspective, the patient’s acceptance of the neurobiological origin of his condition was gradual and initially marked by denial. Insight into the presence of a brain disease therefore evolved from limited to partial over the course of treatment, whereas insight into the alleged criminal behaviour remained largely externalised and contested. Following identification of an organic cause, he declined to engage with the criminal process due to procedural delays and concerns about his life expectancy. Although assisted suicide would have been accessible to the patient, he perceived it as incompatible with his personal values and instead processed his physical and existential suffering through irreverent artistic expression. This pattern reflected a dissociation between emerging medical insight and persistent defensive positioning in the forensic domain, a constellation not uncommon in patients with frontal lobe pathology.

## Discussion

This case highlights several critical dilemmas at the intersection of neuropathology, criminal responsibility and forensic psychiatric care. At its core lies the challenge of determining accountability when criminal acts are driven by severe organic brain disease, when society must balance its obligation to protect against harm while respecting the individual’s rights whose behaviour is shaped by neuropathology. Swiss law, like many jurisdictions, balances public safety with the principle of culpability, often mandating forensic commitment for offenders deemed dangerous despite diminished responsibility. Juridical truth is established only at judgement, with forensic expertise tasked with determining causal links between crime and mental disorder.^15^ Nevertheless, findings of diminished responsibility often collide with institutional limitations, particularly in cases where long-term care facilities are scarce or structurally unsuited for patients with severe neurological decline.

Crucially, this case should not be interpreted as evidence of a direct or deterministic relationship between brain tumours such as glioblastoma and criminal behaviour. Rather, the association observed here is best understood as neurobiologically mediated through the disruption of frontal networks governing executive control, impulse regulation, social cognition and judgement, i.e. mechanisms that have been described in prior neuropsychiatric and lesion-network studies of behavioural dysregulation.^16–19^ It is also crucial to stress that, despite these neurobiological underpinnings, the tumour represents a contextually embedded contributory factor influencing behaviour rather than a single sufficient or generalisable cause of criminality – a fact that appears particularly crucial to stress because biological explanations seem to persistently impact assessment of others’ responsibility more strongly than social determinants,^20^ yet can rarely be fully disentangled.^21^

These tensions are further compounded when organic illness is terminal. In this patient, the progression of glioblastoma not only shaped his behaviour but also curtailed his life expectancy. Placement in a more suitable forensic facility, such as a protected psychiatric unit, could not be secured, because no immediate solution was available during the period of detention. Forensic psychiatry, with its emphasis on detention and legal timelines, faces the challenge of integrating palliative goals of care within judicial timelines, which are often prolonged. Biological fluctuations between deterioration and stability further complicate placement, because pre-trial detention facilities are generally not designed to provide sustained medical or palliative care, allowing only temporary hospital admissions during acute phases. The resulting situation of continued pre-sentence detention despite advanced oncological disease illustrates a profound tension between legal procedure and optimal, humane care. As of 2025, the patient remains alive, still confined under pre-sentence detention, pending completion of judicial instruction.

The patient’s reflections on PAS introduced additional legal and ethical complexity. In Switzerland, PAS is permitted under restrictive conditions.^22^ Whereas patients in psychiatry may access PAS under stringent safeguards, forensic psychiatric patients face further constraints.^23^ The patient’s neurocognitive decline constitutes a substantial obstacle to the lawful and ethical provision of PAS. In accordance with Swiss regulations, the patient would first require a comprehensive assessment to ensure that the underlying condition does not impair or undermine their autonomous decision to pursue assisted suicide. Subsequently, an independent psychiatric evaluation would be necessary to confirm that all remaining legal and ethical criteria, most notably the presence of unbearable suffering and the absence of external pressure, are fully satisfied. In this case, the patient’s glioblastoma poses further problems because it potentially compromises cognitive functions and limits his decisional capacity to make autonomous end-of-life choices.^24^ Given that Swiss regulations place strong emphasis on competence, neurocognitive decline constitutes a significant obstacle to the lawful and ethical implementation of PAS.

Perhaps, however, the most striking dimension of this case lies in the patient’s creative recourse to art. Refusing antidepressant treatment, he turned instead to drawing as a primary coping strategy. His artwork became both a form of protest and a vehicle for self-understanding, fostering resilience and positive self-valorisation. In doing so, he positioned himself within a long history of patients in restrictive psychiatric contexts who transformed their experiences through art. Arguably one of the best known is the afore-mentioned Bernese patient, Adolf Wölfli, whose works were later exhibited at Documenta 5 and are now exhibited in the Kunstmuseum Bern. Other collections, such as the Sammlung Prinzhorn in Heidelberg and the Collection de l’Art Brut in Lausanne, further attest to the therapeutic and cultural value of psychiatric art.^25^ Against this background, the patient’s drawings are more than incidental illustrations of his illness: they represent an act of resilience and a mode of reclaiming agency within the dual constraints of forensic detention and terminal brain disease.

This report is subject to several important limitations. First, as a single-case observation, causal inferences between glioblastoma and criminal behaviour cannot be established and should not be generalised beyond the present clinical context. Second, as discussed above, the behavioural changes described here are most appropriately interpreted as a neurobiological factor embedded in a specific context rather than as evidence of a deterministic relationship between brain tumour and criminality. Third, the retrospective clinical reconstruction of the case, and the jurisdiction-specific aspects particular to the legal framework of Switzerland, limit transferability to other forensic or healthcare systems. Fourth, the patient’s artwork was not submitted for independent expert evaluation but was interpreted within the specific context of his clinical treatment by attending healthcare professionals, introducing a potential risk of interpretive bias and limiting triangulation of the artwork’s psychological and symbolic meaning. Taken together, these limitations underscore the need for a cautious interpretation of the case reported here, as well as the importance of individualised forensic psychiatric assessment.

This case illustrates how severe brain pathology can generate profound forensic and ethical dilemmas, particularly when criminal responsibility and terminal illness intersect. Beyond these challenges, the patient’s reliance on drawing as a coping strategy underscores the frequently overlooked role of art in forensic psychiatry. Recognising such artistic expression not merely as a by-product of disease but as a therapeutic and communicative act invites forensic psychiatry to broaden its perspective, acknowledging art as a vital resource for understanding and supporting patients in similarly complex situations.

## Data Availability

Data availability is not applicable to this article because no new data were created or analysed in this study.
